# Complete genome sequence of *Achromobacter* sp. strain E1, an endophyte from *Zea mays* L. cultivar (Zheng dan 958)

**DOI:** 10.1128/mra.00560-24

**Published:** 2024-08-27

**Authors:** Hao Li, Qingliu Wu, Pugang Yu, Bin Ni

**Affiliations:** 1 State Key Laboratory of Nutrient Use and Management, College of Resources and Environmental Sciences, China Agricultural University, Beijing, China; University of Strathclyde, Glasgow, United Kingdom

**Keywords:** *Achromobacter *sp., plant endophyte, maize, genome analysis

## Abstract

This announcement reports the complete genome sequence of *Achromobacter* sp. strain E1, which was isolated from the root of maize cultivar (Zheng dan 958) grown in Beijing, China. *Achromobacter* sp. strain E1 consists of a single, closed genome consisting of 5,975,307 bp, with GC content of 65.86%.

## ANNOUNCEMENT

*Achromobacter* sp. is a common member of the microbiome of several plants (*Arabidopsis*, *Medicago*, *Brachypodium*, etc.) worldwide (1–6). Recent studies suggested that *Achromobacter* strains were able to utilize plant hormones, such as 1-aminocyclopropane-1-carboxylic acid, indole-3-acetic acid, and salicylic acid, as the sole carbon source (7). We have isolated an *Achromobacter* strain from maize (*Zea mays* L. cultivar “Zheng dan 958”) roots in China.

The 10-cm upper root from a healthy maize grown with natural soil in the greenhouse for 8 weeks was harvested. Sterilized water was used to remove all soil particles. Then, sterilized PBS solution was used to wash the root for three times. All samples were cut into 2 mm pieces and smashed. Smashed root material was diluted 2,000-fold in 10% tryptic soy broth (TSB) medium and pipetted (150 µL) into 96-well plates. Diluted suspension was incubated at room temperature for 2 weeks (8). Pure culture was isolated by continuous streaking on agar plates (50% TSB). Single colonies were inoculated into 50% TSB medium for 12 h. The 16S rRNA gene was amplified with 27F and 1492R, and used to identify the taxonomic identity. BLAST in NT database (20230605) of NCBI identified the closest sequence match as *Achromobacter* sp. (GenBank: MF093189.1).

Genomic DNA of *Achromobacter* sp. strain E1 was extracted using Wizard Genomic DNA Purification Kit (Promega, USA). Genome sequencing was performed by Shanghai Majorbio Bio-Pharm Technology Co., Ltd (Shanghai, China). Genomic DNA was sequenced using a combination of PacBio RS II Single-Molecule Real-Time (SMRT) and Illumina NovaSeq 6000 sequencing platforms. Illumina sequencing libraries were prepared from the sheared fragments using the NEXTflex Rapid DNA-Seq Kit (Bioo Scientific, USA). For Pacific Biosciences sequencing, the g-tubes (Covaris, USA) method was used to shear the genomic DNA to approximately 8–10 kb fragments. The SMRTbell Prep Kit 3.0 (Pacific Biosciences, USA) was used to construct the libraries. A 10-kb insert library was prepared and sequenced on one SMRT cell (Pacific Biosciences, USA).

The genome was determined with PacBio and Illumina (9). All software was deployed using default parameters unless otherwise specified. Raw reads of Illumina were filtered using a fastp software (version 0.20.0) (10). After quality control, a total of 8,130,732 reads of Illumina were obtained. Assembly of Illumina and PacBio reads was performed using Unicycler (version 0.4.8) (11), which includes Pilon (version 1.22) for polishing (12). The genome sequences were circularized using Unicycler, which automatically identified and trimmed the overlapping ends. A complete circular chromosome was successfully assembled without rotation. The details of genome are shown in Table 1. Prodigal (version 2.6.3) (13) was used for coding DNA sequences prediction, tRNA-scan-SE (version 2.0.12) (14) was used for tRNA prediction, Barrnap (https://github.com/tseemann/barrnap) was used for rRNA prediction, and Infernal (version 1.1.5) (15) was used for sRNA prediction.

**TABLE 1:** Sequencing, assembly, and annotation data of *Achromobacter* sp. strain E1

Parameter	Strain E1
Total no. of raw reads (Illumina)	8,392,576
Genome size (bp)	5,975,307
G + C content (%)	65.86
Total no. of reads (PacBio)	73,410
*N*50 value (bp)	8,920
Average read length (bp)	8411.73
Coverage (%)	100
Coding genes in non-redundant protein database (NCBI)	5,425
Coding genes in Swiss-Prot database	4,277
Coding genes in Pfam database	4,822
Coding genes in COG database	4,699
Coding genes in GO database	1,826
Coding genes in KEGG database	4,332

The genome contained 5,433 coding genes, 55 tRNAs, 13 rRNAs, and 19 sRNAs. The coding genes were annotated from non-redundant protein database (NCBI), Swiss-Prot (16), Pfam (17), COG (18), GO, and KEGG databases (19).

## Data Availability

This whole genome shotgun project was deposited in GenBank under the accession number CP146253. Its associated Bioproject and BioSample are PRJNA1078573 and SAMN40006910, respectively. Raw sequence reads were deposited in the GenBank Sequence Read Archive (SRA) under the accession numbers SRR28249060 and SRR28702420. The annotation of genes has been deposited in Zenodo (https://doi.org/10.5281/zenodo.11057739).
